# Peroxisome Proliferator-Activated Receptor Alpha Target Genes

**DOI:** 10.1155/2010/612089

**Published:** 2010-09-26

**Authors:** Maryam Rakhshandehroo, Bianca Knoch, Michael Müller, Sander Kersten

**Affiliations:** ^1^Nutrition, Metabolism and Genomics Group, Division of Human Nutrition, Wageningen University, Bomenweg 2, 6703 HD Wageningen, The Netherlands; ^2^Food, Metabolism & Microbiology, Food & Textiles Group, AgResearch, Palmerston North 4442, New Zealand; ^3^Institute of Food, Nutrition & Human Health, Massey University, Tennent Drive, Palmerston North 4442, New Zealand

## Abstract

The peroxisome proliferator-activated receptor alpha (PPAR*α*) is a ligand-activated transcription factor involved in the regulation of a variety of processes, ranging from inflammation and immunity to nutrient metabolism and energy homeostasis. PPAR*α* serves as a molecular target for hypolipidemic fibrates drugs which bind the receptor with high affinity. Furthermore, PPAR*α* binds and is activated by numerous fatty acids and fatty acid-derived compounds. PPAR*α* governs biological processes by altering the expression of a large number of target genes. Accordingly, the specific role of PPAR*α* is directly related to the biological function of its target genes. Here, we present an overview of the involvement of PPAR*α* in lipid metabolism and other pathways through a detailed analysis of the different known or putative PPAR*α* target genes. The emphasis is on gene regulation by PPAR*α* in liver although many of the results likely apply to other organs and tissues as well.

## 1. Introduction

Nutrient metabolism and energy homeostasis are tightly controlled by numerous regulatory systems involving specific transcription factors. The peroxisome proliferator-activated receptors (PPARs) are ligand-activated transcription factors that belong to the superfamily of nuclear hormone receptors and play an important role in nutrient homeostasis [1–3]. Three different PPAR subtypes are known: PPAR*α*, PPAR*β*/*δ*, and PPAR*γ*. All PPARs share the same molecular mode of action via formation of heterodimers with the nuclear receptor RXR, followed by binding to specific DNA-response elements in target genes known as peroxisome proliferator response elements (PPREs). PPREs are characterized by a common core sequence consisting of a direct repeat of the consensus sequence AGGTCA interspaced by a single nucleotide [1, 4]. Expression of PPAR*α* and PPAR*β*/*δ* is found ubiquitously, whereas PPAR*γ* is mainly expressed in adipose tissue, macrophages, and colon [5, 6]. Activation of transcription by PPARs is dependent on a number of different steps including ligand binding to PPAR, binding of PPAR to the target gene, removal of corepressors and recruitment of coactivators, remodeling of the chromatin structure, and finally facilitation of gene transcription [7]. This paper will focus exclusively on PPAR*α*. 

PPAR*α* was first discovered in the early 1990s and since then has been identified as the master regulator of hepatic lipid metabolism [8]. In addition, PPAR*α* has been shown to govern glucose metabolism, lipoprotein metabolism, liver inflammation, amino acid metabolism, and hepatocyte proliferation (specifically in rodents). Synthetic agonists of PPAR*α* lower plasma triglycerides and raise plasma high-density lipoprotein (HDL) levels and are thus used clinically in the treatment of dyslipidemia [2, 9–11].

In recent years, the advent of microarray technology has allowed the study of whole genome expression profiles. Accordingly, a wealth of new information has become available about the role of specific transcription factors in regulation of gene expression. Combined with data collected using more established methods, microarray has permitted the generation of a comprehensive picture of the impact of PPAR*α* on gene expression, thereby providing key insight into the functional role of PPAR*α*. The present paper is aimed at providing a detailed and updated overview of PPAR*α* target genes in different biological processes and to highlight possible differences between mouse and human. 

Although the presence of a functional PPRE is often used as a criteria for designating direct PPAR*α* target genes, we did not apply this criteria very stringently in our analysis as the in vivo functionality of most of the identified PPREs remains uncertain. Recent studies indicate that the standard approach to screen for PPREs in the 1-2 kb region upstream of the transcriptional start site (TSS) is at odds with accumulating evidence that PPARs often bind quite distant from the TSS [12–14]. In those cases, contact with the basal transcription machinery is expected to be established via DNA looping. Thus, the absence of a PPRE in the 1-2 kb region upstream of the TSS cannot be used as a criterion to disqualify target genes. Other aspects that need to be taken into account include correspondence in gene function with better established PPAR targets and the timing of gene induction following PPAR*α* activation.

## 2. PPAR*α* Tissue Expression Profile in Mouse and Human

**Figure 1:**
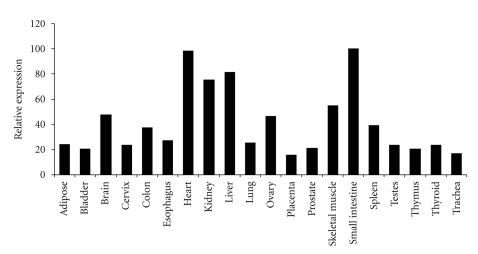
*Expression profile of* *P* *P* *A* *R* *α* *in human tissues.* The FirstChoice Human Total RNA Survey Panel (Ambion) was reverse transcribed and used for qPCR using primers specific for human PPAR*α*. Expression levels are expressed relative to small intestine, which showed the highest expression level (100%).

High expression levels of PPAR*α* expression are found in liver and specifically in the parenchymal cell population. Expression of PPAR*α* in nonparenchymal liver cells such as Kupffer cells is much lower [15, 16]. Other tissues with high PPAR*α* mRNA levels are heart, kidney, intestine, and brown adipose tissue, all of which are characterized by an elevated rate of fatty acid catabolism [17]. PPAR*α* expression has also been detected in immune cells such as the peripheral blood mononuclear cell population, and specifically in T-cells and macrophages [18–22]. Evidence suggests that mice and humans share similar PPAR*α* tissue expression profiles [6, 17] (Figure 1). In the past, the importance of PPAR*α* in human liver was questioned based on data showing approximately 10-fold lower PPAR*α* mRNA levels in human liver compared with mouse liver [23]. A recent study using more advanced methodology revealed similar PPAR*α* expression in mouse and human liver and in mouse and human hepatocytes, thus strongly arguing against this notion [24]. Given that PPAR*α* has been most extensively studied in liver, most of the information on PPAR*α* target genes presented here relates to hepatic gene regulation.

## 3. PPAR*α* Structure in Mouse and Human

Analogous to other nuclear receptor superfamily members, PPAR*α* has a domain structure consisting of an N-terminal activating function-1 (AF-1) domain, a central DNA-binding domain (DBD), and a C-terminal ligand-binding domain (LBD) [25, 26]. The N-terminal domain can be phosphorylated leading to changes in transcriptional activity and even ligand binding of the receptor [27]. The DBD is responsible for physical interaction with DNA and allows PPAR*α* to bind to specific PPREs as a heterodimer with RXR [28]. The LBD harbors the ligand-binding pocket crucial for dimerization with RXR and contains the activating function-2 involved in physical interactions with coregulatory proteins [7, 29, 30]. Comparison of human and murine PPAR*α* shows 85% identity at the nucleotide level and 91% identity at the amino acid level. Data have indicated that there is some genetic heterogeneity in the functional coding sequence of human PPAR*α* that translates into functional differences in receptor activity. One identified variant of the human PPAR*α* gene produces a protein that is mutated within the PPAR*α* DNA-binding domain. This L162V gene variant exhibits greater ligand-induced activity compared to the wild-type receptor [31, 32]. While there is some evidence for a link between the L162V polymorphism and metabolic parameters such as plasma lipid levels, these correlations are not always found [32–37]. Interestingly, the effect of L162V polymorphism has been suggested to be modulated via gene-drug and gene-nutrient interactions [38–40]. The V227A polymorphism was found in Japanese population and has been associated with altered serum lipid levels and nonalcoholic fatty liver disease [41–44]. In addition to polymorphic variants, a truncated splice variant of human PPAR*α* has been described that negatively interferes with wild-type PPAR*α* activity [45].

## 4. PPAR*α* Ligands

PPAR*α* serves as receptor for a structurally diverse set of compounds. The most important class of synthetic PPAR*α* ligands is the fibrates, including gemfibrozil, bezafibrate, clofibrate, fenofibrate, and Wy14643 [2, 9–11, 46]. This class of drugs is used in the treatment of dyslipidemia primarily associated with type 2 diabetes mellitus. In addition, PPAR*α* is activated by plasticizers, insecticides, and other rodent hepatic carcinogens. Natural ligands of PPAR*α* include a variety of fatty acids as well as numerous fatty acid derivatives and compounds showing structural resemblance to fatty acids, including acyl-CoAs, oxidized fatty acids, eicosanoids, endocannabinoids, and phytanic acid [47–53]. Endogenous ligand activation of PPAR*α* in liver was initially suggested to occur primarily during fasting as large amounts of free fatty acids are released into the bloodstream and enter the liver [54, 55]. However, compelling evidence indicates that hepatic PPAR*α* is not activated by plasma free fatty acids, whereas it can be activated by dietary fatty acids and fatty acids generated via de novo lipogenesis [56–60]. Recently, it was shown that the effects of dietary unsaturated fatty acids on hepatic gene expression are almost exclusively mediated by PPAR*α* and mimic the effect of synthetic PPAR*α* agonists [61].

## 5. PPAR*α* and Hepatic Lipid Metabolism

**Figure 2:**
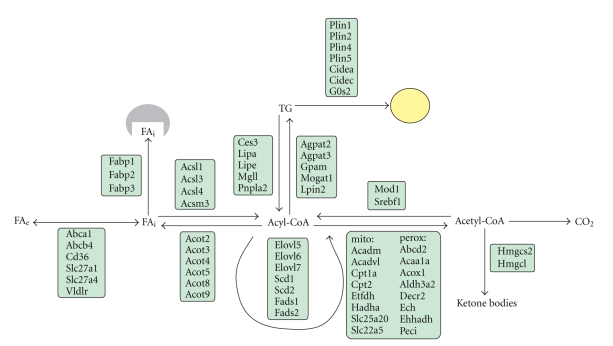
Schematic representation of PPAR*α* target genes in different aspects of hepatic lipid metabolism.

Regulation of lipid metabolism is mainly coordinated by liver, which actively metabolizes fatty acids as fuel and continuously produces very low-density lipoproteins (VLDLs) particles to provide a constant supply of fatty acids to peripheral tissues. Disturbances in these pathways are the basis for hepatic steatosis and alterations in plasma lipoprotein levels. Many aspects of hepatic lipid metabolism are under control of PPAR*α*, including fatty acid uptake through membranes, fatty acid activation, intracellular fatty acid trafficking, fatty acid oxidation and ketogenesis, and triglyceride storage and lipolysis (Figure 2). It has been suggested that part of the effect of PPAR*α* on hepatic ketogenesis may be mediated by induction of the PPAR*α* target fibroblast growth factor 21 [90–92]. A detailed discussion of the specific genes within the various lipid metabolic pathways that are targeted by PPAR*α* is provided below (Table 1).

### 5.1. Peroxisomal Fatty Acid *β*-Oxidation

The first link between PPAR*α* and fatty acid catabolism was established by the identification of the Acyl-CoA oxidase gene, encoding the rate-limiting enzyme in peroxisomal long-chain fatty acid oxidation, as a direct PPAR*α* target gene [98, 172]. Peroxisomes are known to be involved in many aspects of lipid metabolism, including synthesis of bile acids and plasmalogens, synthesis of cholesterol and isoprenoids, alpha-oxidation, glyoxylate and H_2_O_2_ metabolism, and beta-oxidation of very-long-straight-chain or branched-chain acyl-CoAs. The beta-oxidation of straight-chain acyl-CoAs starts with a reaction catalyzed by acyl-CoA oxidase 1 (Acox1) followed by one of two enzymes carrying both enoyl-CoA-hydratase and 3-hydroxyacyl-CoA dehydrogenase activity (L-bifunctional enzyme, Ehhadh; D-bifunctional enzyme, Hsd17b4) and finally peroxisomal 3-ketoacyl-CoA thiolase (Acaa1a, Acaa1b). All genes mentioned above represent PPAR*α* targets [24, 55, 62, 68, 70, 76, 98, 99, 101–104]. Additionally, genes involved in peroxisomal fatty acid uptake (Abcd2 and Abcd3), conversion of fatty acid to acyl-CoA (Crot), and numerous thioesterases (Acots) that convert acyl-CoAs back to fatty acids have been reported to be regulated by PPAR*α* [24, 62, 74, 96, 97]. Activation of PPAR*α* using synthetic agonists is known to cause massive proliferation of peroxisomes in rodents via induction of a large set of genes encoding peroxisomal fatty acid oxidation enzymes, as well as genes involved in peroxisomal biogenesis (Pex genes). Chronic exposure to these so-called peroxisome proliferators can also induce liver cancer in rodents [173]. In contrast, activation of PPAR*α* in humans does not seem to induce hepatocellular carcinomas, suggesting a species specific response to PPAR*α* activation. Initially, it was believed that the differential response was due to the lack activation of Acox1 and other peroxisomal genes by PPAR*α* in humans [71, 174, 175]. However, recent data indicate that PPAR*α* is able to induce a significant number of genes involved in peroxisomal fatty acid oxidation in human primary hepatocytes, including Acox1 [24]. Also, PPAR*α*-mediated induction of the Pex11a gene involved in peroxisome proliferation is observed in both species [24].

### 5.2. Mitochondrial Fatty Acid *β*-Oxidation

The crucial role of PPAR*α* in mitochondrial fatty acid oxidation is illustrated by the phenotype of fasted PPAR*α* ^−/−^ mice, which exhibit hypoketonemia, hepatic steatosis, and elevated plasma free fatty acid levels [54, 55, 176]. It is now evident that virtually every enzymatic step within the fatty acid oxidative pathway is under control of PPAR*α*. Specifically, PPAR*α* induces genes controlling fatty acid import into the mitochondria (Cpt1, Cpt2, Slc25a20, Slc22a5), as well as the major enzymes within the *β*-oxidation pathway, including various acyl-CoA dehydrogenases (Acad, step 1), mitochondrial trifunctional enzyme (Hadh, step 2–4), and genes involved in *β*-oxidation of unsaturated fatty acid (Dci, Decr) [24, 54, 55, 62, 63, 65, 68, 70, 74, 76–86].

Additionally, synthesis of ketone bodies via mitochondrial HMG-CoA synthase (Hmgcs2) and HMG-CoA lyase (Hmgcl) is governed by PPAR*α* [24, 62, 93–95], as is the expression of genes encoding electron transferring flavoprotein and the corresponding dehydrogenase (Etfa, Etfb, Etfdh) [24, 62]. The latter proteins mediate the transfer of electrons from Acyl-CoA dehydrogenases to the membrane-bound electron transfer flavoprotein ubiquinone oxidoreductase, allowing further entry into the oxidative phosphorylation pathway [177, 178]. Finally, PPAR*α* induces uncoupling proteins Ucp2 and Ucp3, which have been proposed to function as an outward transporter of nonesterified fatty acid anions from the mitochondrial matrix [24, 62, 87–89].

### 5.3. Microsomal Fatty Acid *ω*-Hydroxylation

Cyp4A enzymes are members of the cytochrome P450 monoxygenase superfamily and catalyze microsomal *ω*-hydroxylation of fatty acids [106, 179]. Studies using PPAR*α* ^−/−^ mice have shown that hepatic expression of Cyp4a genes is almost completely dependent on PPAR*α* (Cyp4a10, Cyp4a12, Cyp4a14 in mice, Cyp4a1, Cyp4a3 in rat, Cyp4a11 in human) [55, 62, 74, 84, 106–111]. Furthermore, expression is extremely sensitive to PPAR*α* ligand activation, indicating that Cyp4a genes may serve as PPAR*α* marker genes. Although previous studies performed in human primary hepatocytes could not show regulation of Cyp4a by human PPAR*α*, our microarray data revealed significant induction of Cyp4a11 by Wy14643 in primary human hepatocytes [24, 68, 180, 181]. *ω*-hydroxylation of saturated and unsaturated fatty acids may lead to the generation of high-affinity PPAR*α* ligands, including hydroxyeicosatetraenoic acids (HETEs), thus creating a positive feedback loop [182]. Alternatively, induction of *ω*-oxidation by PPAR*α* has been suggested to promote the degradation of the PPAR*α* agonist leukotriene B4 as part of a feedback mechanism aimed at controlling the duration of the inflammatory response [53].

### 5.4. Hepatic Lipogenesis

Whereas PPAR*α* is mostly known for its ability to induce fatty acid oxidation, growing evidence points to a role of PPAR*α* in regulation of lipogenesis. A functional PPRE was identified in the promoter of a limited number of lipogenic genes including Δ6 desaturase (Fads2), malic enzyme (Mod1), phosphatidate phosphatase (Lpin2), and Δ9 desaturase (Scd1) [56, 115–117]. Gene expression profiling showed that chronic in vivo treatment of mice with PPAR*α* agonist causes the upregulation of a large set of lipid biosynthetic genes [62]. However, regulation is much less pronounced in primary hepatocytes, suggesting an indirect mechanism. Consistent with this notion, induction of lipogenic genes by chronic PPAR*α* activation was completely abolished in SREBP1^−/−^ mice [183]. The effect of PPAR*α* agonists on SREBP targets has been attributed to increased activation of SREBP1c via enhanced proteolytic cleavage [112]. Such a mechanism may also lead to increased SREBP1 mRNA via an autoloop regulatory circuit [184]. Alternatively, it is possible that PPAR*α* is recruited to promoters of SREBP targets and stimulates SREBP activity [12]. Interestingly, in rat FAO hepatoma cells, it was found that PPAR*α* activation reduced expression of lipogenic genes, including Fasn, Gpam, and SREBP1c, while Insig1 expression was increased by PPAR*α* [185]. The reason for the discrepancy is not clear.

In contrast to de novo fatty acid and cholesterol synthesis, synthesis of triglycerides may be directly targeted by PPAR*α*. Several genes within these pathways are upregulated by PPAR*α* activation, including Gpam, various Agpat genes, Mogat1, Dgat1, and Lpin2 [24, 62, 65, 112]. Induction of genes involved in triglyceride synthesis from fatty acids may reflect a broader role of PPAR*α* in the hepatic response to fasting aimed at neutralizing large amounts of incoming adipose tissue-derived free fatty acids.

### 5.5. Fatty Acid Uptake and Binding

Before they can be metabolized in the liver, fatty acids have to be transferred across the cell membrane. Several proteins are involved in fatty acid transport across the plasma membrane, a number of which carry both fatty acid transporter and acyl-CoA synthetase activity. Studies have shown that the fatty acid transport proteins Slc27a1, Slc27a2, and Slc27a4 are upregulated by PPAR*α* in liver [24, 62, 64–68, 101]. 

Slc27a1 is not expressed and not regulated by PPAR*α* in isolated primary hepatocytes, suggesting regulation occurs in liver macrophages (Kupffer cells). So far, the only fatty acid transporter for which a PPAR response element has been identified is Slc27a1. PPAR*α* agonists also markedly induce hepatic expression of the fatty acid transporter/scavenger receptor Cd36, which is expressed in various liver cell types [24, 62–64]. Additionally, expression of numerous acyl-CoA synthetases is induced by PPAR*α* [24, 62, 63, 70–73]. Currently, limited information is available about the cellular localization and the structure/function relationship of acyl-CoA synthetase enzyme [186]. 

The Fabp gene family comprise a group of high-affinity intracellular fatty acid-binding proteins. Interestingly, Fabp1 was one of the first PPAR*α* target genes identified [74, 75, 187, 188]. Recent studies indicate that Fabp1 may be involved in partitioning of FA to specific lipid metabolic pathways [189]. Other Fabp genes induced by PPAR*α* activation in mouse liver include Fabp2, Fabp3, Fabp4, and Fabp5 [24, 62, 63]. Induction of Fabp4 (A-FABP, aP2) upon PPAR*α* activation likely occurs via its expression in Kupffer cells. Fabp4 expression in hepatocytes is correlated with acquisition of a steatotic phenotype concurrent with upregulation of PPAR*γ* mRNA [190].

### 5.6. Lipases and Lipid Droplet Proteins

PPAR*α* ^−/−^ mice exhibit elevated hepatic TG accumulation, especially under fasting conditions [54, 191, 192]. Conversely, treatment with PPAR*α* agonists lowers hepatic triglyceride levels in models of hepatic steatosis and can prevent the fasting-induced increase in liver TG [193, 194]. The antisteatotic effect of PPAR*α* has mainly been attributed to stimulation of fatty acid oxidation, which would decrease the availability of fatty acids for TG storage. 

Recently, hepatic lipid droplets were shown to be targeted by autophagy, which ultimately leads to TG hydrolysis via lysosomal acid hydrolase (Lipa). Which other lipases importantly contribute to intracellular lipolysis of hepatic TG stores remains unclear, but lipases active in adipocytes likely play a role, including Ces3, Lipe, Pnpla2, Mgll, and perhaps Pnpla3 [195–200]. With the exception of Pnpla3, all of the above genes are induced by short-term treatment with PPAR*α* agonist in mouse hepatocytes. Regulation of Pnpla2 was also observed in human hepatocytes. Pnpla2 and Lipe were previously classified as direct target genes of PPAR*γ* in adipose tissue, suggesting that they are direct target of PPAR*α* as well [201, 202]. Thus, apart from induction of fatty acid oxidation, PPAR*α* activation may also decrease hepatic TG storage by stimulating the TG hydrolysis pathway.

Lipid droplets are coated with one or more members of the perilipin family of proteins: perilipin (Plin1), Adrp/adipophilin (Plin2), Tip47 (Plin3), S3-12 (Plin4), and Oxpat/Lsdp5 (Plin5). Adrp and Lsdp5 have been identified as target genes of PPAR*α* in liver [120, 124]. A recent study suggests that Adrp could serve as potential mediator of the effect of PPAR*α* on VLDL production. Adrp induction by PPAR*α* may diminish VLDL production by favoring fatty acids storage in cytosolic lipid droplets rather than directing through VLDL assembly [203]. Besides Adrp, expression of S3-12 and perilipin, which are known as PPAR*γ* target genes in adipose tissue, is induced by PPAR*α* agonist in human hepatocytes [24, 204]. Perilipin expression in human liver is correlated with development of steatotic liver [205]. 

Two recently identified lipid droplet-associated proteins that are not part of the perilipin family are Cidec (FSp27) and Cidea [206, 207]. Both proteins promote TG accumulation and are targets of PPAR*γ* in adipocytes [208, 209]. In addition, they are regulated by PPAR*α* in mouse liver, although the kinetics of induction of the two genes seems to be quite different [121]. Cidec but not Cidea upregulation by PPAR*α* agonist could be confirmed in human primary hepatocytes [24]. 

Interestingly, the G(0)/G(1) switch gene 2 (G0s2) was recently identified as an inhibitor of Pnpla2 activity and located to lipid droplets in adipocytes stimulated with *β*-adrenergic receptor agonist [122]. Previously, G0s2 was shown to be a direct PPAR*α* target gene in mouse liver and PPAR*γ* target in adipocytes [123]. Whether G0s2 associates with lipid droplets in hepatocytes remains to be further investigated. Similar to the induction of triglyceride synthesis, regulation of numerous lipid droplet proteins by PPAR*α* reflects a broader role of PPAR*α* in the hepatic response to fasting aimed at deflecting large amounts of incoming adipose tissue-derived free fatty acids towards storage in lipid droplets.

## 6. PPAR*α* and Lipoprotein Metabolism

Clinical studies in humans have provided ample evidence that fibrate drugs effectively lower fasting plasma triglycerides (TG) and raise plasma HDL [210–213]. At the molecular level, fibrates act as synthetic agonist for PPAR*α*, indicating an important role of PPAR*α* in the control of lipoprotein metabolism. PPAR*α* lowers plasma TG in part by reducing very low-density lipoprotein (VLDL) production [194]. Traditionally, this effect of PPAR*α* was ascribed to induction of genes involved in fatty acid oxidation and the concomitant reduction in lipid availability for VLDL production. However, this paper has made it evident that in addition to its role in fatty acid catabolism, PPAR*α* influences multiple aspects of intracellular lipid trafficking and metabolism, some of which may oppose hepatic TG lowering. Furthermore, expression of Mttp, which is involved in the lipidation of apoB100 to form a nascent VLDL particle, is positively regulated by PPAR*α* [214]. Thus, the precise target genes underlying the suppressive effect of PPAR*α* agonist on hepatic VLDL production remain to be fully elucidated.

In addition to suppressing VLDL production, PPAR*α* agonists are known to stimulate clearance of TG-rich lipoproteins [194]. Clearance of TG-rich lipoproteins VLDL and chylomicrons is mediated by the enzyme lipoprotein lipase (LPL), which is attached to the capillary endothelium of muscle and adipose tissue. Expression of Lpl in liver is restricted to Kupffer cells and upregulated by PPAR*α* agonists [140, 215]. In contrast, no evidence is available indicating a stimulatory effect of PPAR*α* on Lpl expression in heart and skeletal muscle, which account for the major share of plasma TG clearance [140, 216]. LPL activity is mostly regulated posttranslationally via altered secretion from liver of LPL-modulating factors, including apolipoprotein C-III (Apoc3), apolipoprotein A-V (Apoa5), Angiopoietin-like protein 3 (Angptl3), and Angiopoietin-like protein 4 (Angptl4). Firstly, PPAR*α* agonists downregulate the expression of LPL inhibitor APOC3, supposedly via mechanisms involving the transcription factors REV-ERB*α*, HNF4*α*, or FOXO1 [137, 217–219]. Secondly, PPAR*α* agonists increase hepatic expression and plasma levels of APOA5, which is a positive regulator of LPL [220]. A functional PPAR responsive element has been identified in the promoter of the human APOA5 gene, classifying APOA5 as a direct PPAR*α* target gene [135, 136]. Thirdly, PPAR*α* upregulates hepatic expression and plasma levels of Angptl4, which acts as inhibitor of LPL activity by converting active LPL dimers to inactive monomers [126]. The DNA response element conferring PPAR regulation was located to intron 3 of the Angptl4 gene [127]. Finally, PPAR*α* stimulates hepatic expression of the VLDL receptor (Vldlr) [24, 62]. The functional significance of Vldlr regulation in liver is unclear, as Vldlr is most highly expressed in adipose tissue, heart, and skeletal muscle, where it plays an auxiliary role in plasma TG hydrolysis by LPL. Recently, Vldlr was shown to be under control of PPAR*γ* in adipocytes [221]. Thus, it appears that both pro- and antilipolytic pathways are activated by PPAR*α*. Under conditions of pharmacological PPAR*α* activation, the prolipolytic actions of PPAR*α* dominate, as illustrated by the stimulation of plasma TG clearance.

PPAR*α* agonists raise plasma HDL levels in humans, which is most likely achieved via species specific mRNA induction of apolipoprotein A-I (Apoa1) and A-II (Apoa2) [128, 175, 222–224]. Apoa1 gene expression is not induced by PPAR*α* in rodents due to the presence of disabling mutations within the PPAR-response element [129]. In fact, PPAR*α* activation in mouse downregulates Apoa1 mRNA expression and plasma concentrations through an indirect pathway involving the PPAR*α*-dependent induction of the nuclear receptor REV-ERB*α*, a negative regulator of transcription [129, 130, 225]. 

The impact of PPAR*α* in HDL metabolism likely extends beyond regulation of apolipoproteins. Evidence suggests that both PPAR*α* and PPAR*β*/*δ* stimulate expression of endothelial lipase (Lipg) in liver [62, 226]. Endothelial lipase mainly carries phospholipase activity and its overexpression was shown to significantly reduce plasma HDL cholesterol levels [227–229]. Since Lipg is expressed in endothelial cells, macrophages, and hepatocytes, regulation of hepatic Lipg by PPAR*α* and PPAR*β*/*δ* may be mediated by different cell types. In as much as PPAR*α* agonists raise plasma HDL levels, the physiological relevance of Lipg induction by PPAR*α* remains to be established. 

In our recent publication, the PPAR*α* agonist Wy14643 modestly induced hepatic lipase (Lipc) expression in primary human hepatocytes [24]. Hepatic lipase exhibits both phospholipase and triglyceride hydrolase activity and hydrolyzes triglycerides and phospholipids of chylomicron remnants, IDL, and HDL [230]. Whether Lipc represents a direct target gene of PPAR*α* in human remains unclear. Other genes involved in lipoprotein metabolism that are regulated by PPAR*α* include phosphatidylcholine transfer protein (Pctp). Induction of Pctp mRNA by PPAR*α* is conserved in primary human hepatocytes [24]. Pctp encodes a steroidogenic acute regulatory-related transfer domain protein that binds with high affinity to phosphatidylcholines. In a recent publication, a role for Pctp in the metabolic response to PPAR*α* was proposed [231]. Overall, it is evident that PPAR*α* governs multiple aspects of plasma lipoprotein metabolism.

## 7. PPAR*α* and Glucose/Glycerol Metabolism

Although PPAR*α* has mostly been linked to fatty acid metabolism, studies in mice have yielded considerable evidence for a role of PPAR*α* in hepatic glucose metabolism. Indeed, fasted PPAR*α* ^−/−^ mice display severe hypoglycemia [54, 55, 176]. Several mechanisms may account for the hypoglycemia, including decreased hepatic glucose production and increased peripheral glucose utilization. Genes involved in gluconeogenesis that have been identified as PPAR*α* targets include phosphoenolpyruvate carboxykinase (Pck1), pyruvate carboxylase (Pcx), and lactate dehydrogenase A [62]. Interestingly, regulation of Pck1 by PPAR*α* was only observed in human hepatocytes [24]. Pyruvate carboxylase was identified as direct target of PPAR*γ* in adipocytes [232].

PPAR*α* was shown to have a specific role in the metabolic conversion of glycerol in liver by directly upregulating expression of genes such as Gpd1, Gpd2, Gyk, Aqp3, and Aqp9 [151]. Besides governing glucose production, PPAR*α* may also alter glucose utilization in numerous tissues via induction of pyruvate dehydrogenase kinase isoform 4 (Pdk4) [153, 154, 233–236]. Pdk4 phosphorylates and inactivates pyruvate dehydrogenase, thereby limiting carbon flux through glycolysis. Synthesis of glycogen is also affected in PPAR*α* ^−/−^ mice, which may be mediated in part via defective regulation of Gys2 [152]. It is noteworthy that in contrast to studies in mice, human trials generally do not support an effect of PPAR*α* activation on plasma glucose levels. Consistent with these data, it was found that upregulation of genes involved in the glycolysis/gluconeogenesis pathway by Wy14643 was uniquely observed in mouse hepatocytes and not human hepatocytes [24].

## 8. PPAR*α* and Hepatic Cholesterol/Bile Metabolism

It has been demonstrated that PPAR*α* activation increases efflux of cholesterol to HDL. Formation of nascent HDL is mediated by Abca1-dependent lipidation of newly-secreted Apoa1. Expression of Abca1 is upregulated by PPAR*α* agonists in both human and mouse hepatocytes, as well as in mouse intestine [24, 237]. Presently, it is not clear if this effect of PPAR*α* activation is mediated via LXR*α*, as was shown previously in macrophages [21]. Other genes involved in cholesterol uptake and transport that were shown to be under control of PPAR*α* include Abcg5, Abcg8, Cav1, Npc1, and Rab9 [24, 62, 144].

While PPAR*α* is known to govern specific genes involved in bile acid synthesis, the overall impact on bile acid homeostasis remains somewhat ambiguous. Expression of Cyp7a1, which represents the rate-limiting enzyme in bile acid synthesis, is markedly downregulated in PPAR*α* ^−/−^ mice in fasting condition [62]. Paradoxically, synthetic PPAR*α* agonists reduce Cyp7a1 expression in both mice and human [145, 238–240]. In agreement with the latter observation, fibrate treatment leads to decreased bile acid synthesis. To what extent the changes in Cyp7a1 expression reflect direct regulation by PPAR*α* is unclear as PPAR*α* also influences the expression of other nuclear hormone receptors involved in regulation of Cyp7a1 such as FXR and LXR. It has also been suggested that PPAR*α* can antagonize LXR signaling and LXR-dependent activation of Cyp7a1 gene promoter [241–243].

Other genes involved in bile acid synthesis that are regulated by PPAR*α* include Cyp27a1 which is downregulated by PPAR*α* agonists in a PPAR*α*-dependent manner [145], and Cyp8b1 which is upregulated by PPAR*α* [62, 148]. Recently, CYP7b1 expression was shown to be suppressed by PPAR*α* in a sex-specific manner, which was shown to occur via sumoylation of the LBD of PPAR*α* [244]. Finally, PPAR*α* stimulates expression of the hepatobiliary phospholipid transporter Abcb4 [24, 62, 94, 144].

## 9. PPAR*α* and Amino Acid Metabolism

 Accumulating evidence supports a role for PPAR*α* in regulation of amino acid and urea metabolism [159, 160, 162, 245]. Studies in mice have shown that PPAR*α* governs metabolism of amino acids by suppressing expression of genes involved in transamination (Aspartate amino transferase (Got1), Alanine amino transferase (Gpt), Alanine glyoxylate aminotransferase (Agtx2), and deamination (Glutaminase (Gls)), as well as numerous genes that are part of the urea cycle (Cps1, Otc, Ass1, and Asl) [160, 161, 245]. In agreement with these data, PPAR*α* ^−/−^ mice exhibit increased plasma urea levels [160]. Several of the above genes were also downregulated by PPAR*α* agonist in primary human hepatocytes, suggesting that regulation of nitrogen metabolism by PPAR*α* is at least partially conserved between mice and human [24]. 

At the present time, the mechanism behind downregulation of nitrogen metabolism by PPAR*α* remains elusive. It has been proposed that PPAR*α* may modulate the activity of other transcription factors that are directly involved in amino acid homeostasis, including HNF4*α* and C/EBP*α* [160]. However, concrete evidence supporting such a mechanism is lacking.

Whereas PPAR*α* activation decreases hepatic aminotransferase expression in mice, PPAR*α* agonists were shown to increase expression of Gpt in human hepatocytes and HepG2 cells, which occurred via direct regulation of the gene promoter [161, 246]. The observed increase in plasma alanine amino transferase activity in patients treated with fibrates may thus be related to direct regulation of Gpt transcription, rather than drug-induced liver injury.

## 10. PPAR*α* and Inflammation

Besides regulating numerous metabolic pathways, PPAR*α* also governs inflammatory processes, which is mainly achieved by downregulating gene expression via a mechanism generally referred to as transrepression. The first clue towards anti-inflammatory effects of PPAR*α* came from the observation that PPAR*α* ^−/−^ mice exhibit a prolonged inflammatory response in the ear swelling test [53]. The anti-inflammatory effects of PPAR*α* are likely explained by interference of PPAR*α* with the activity of many proinflammatory transcription factors including signal transducer and activator of transcription (Stat), Activator protein-1 (AP-1), and NF-*κ*B [247]. Specifically, it has been shown that activated PPAR*α* binds to c-Jun and to the p65 subunit of NF-*κ*B, thereby inhibiting AP-1- and NF-*κ*B- mediated signaling [248]. Additionally, PPAR*α* induces the inhibitory protein I*κ*B*α*, which normally retains NF-*κ*B in a nonactive form, leading to suppression of NF-*κ*B DNA-binding activity [168]. Suppression of fibrinogen gene expression by PPAR*α* activation is likely mediated by interference with the transcription factor CAATT/enhancer-binding protein (C/EBP) via sequestration of the coactivator glucocorticoid receptor-interacting protein 1/transcriptional intermediary factor 2 (GRIP1/TIF2) [166]. Finally, recent data indicate that activated PPAR*α* may downregulate gene expression by causing the loss of STAT1 and STAT3 binding to DNA [12]. 

Specific genes downregulated by PPAR*α* include a number of acute phase genes such as fibrinogen, serum amyloid P-component, lipocalin 2, metallothioneins, and serum amyloid A2, which were shown to be suppressed by the PPAR*α* agonist Wy14643 in wild-type mice but not PPAR*α* ^−/−^ mice [163]. Similarly, in humans fenofibrate treatment has been shown to decrease plasma levels of several acute phase proteins including C-reactive protein, fibrinogen-*α* and -*β* and interleukin 6 [166, 249, 250]. With the exception of the sIl-1 receptor antagonist and Vanin-1, to our knowledge no inflammatory genes have been identified as direct positive targets of PPAR*α* [163].

**Figure 3:**
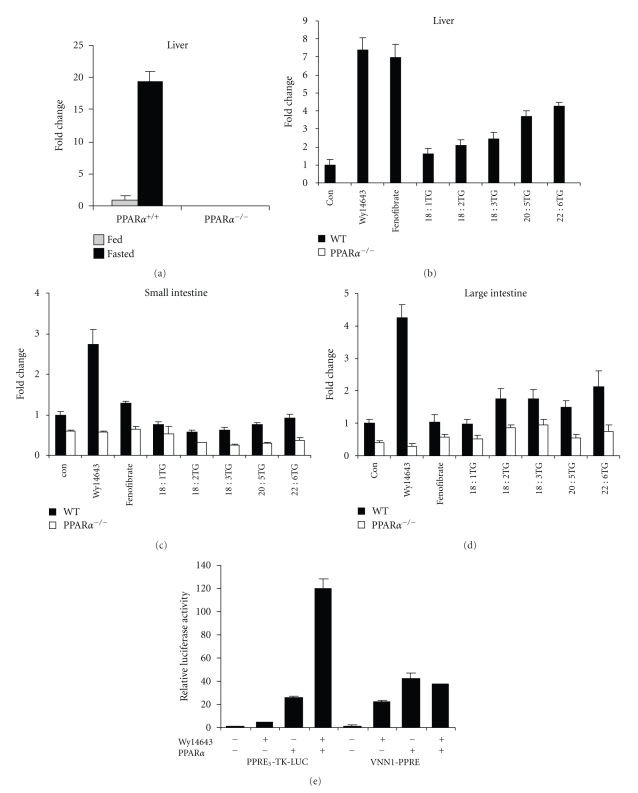
*Vanin-1 likely represents a direct* *P* *P* *A* *R* *α* *target gene.* (a) Vnn1 expression in livers of ad libitum fed and 24 h fasted wildtype and PPAR*α* ^−/−^ mice. (b) Vnn1 expression in liver, (c) small intestine, and (d) large intestine of wildtype and PPAR*α* ^−/−^ mice 6 h after administration of a single oral dose of Wy14643 (4 mg), fenofibrate (4 mg), and synthetic triglycerides triolein, trilinolein, trilinolenin, trieicosapentaenoin, or tridocosahexaenoin (400 mL). (e) HepG2 cells were transiently transfected with reporters (PPRE)3-TK-LUC or PPRE-Vnn1-LUC (PPRE present in intron 3-4 of the Vnn1 gene cloned into pGL3-promoter) and PPAR*α* expression plasmid (pSG5). After transfection, cells were treated with WY14643 (50 *μ*M) for 24 hours followed by determination of luciferase and *β*-galactosidase activities in the cell lysates. Luciferase activities were normalized to *β*-galactosidase, and the relative luciferase activity of the cells treated with DMSO was set to 1. Error bars represent SEM.

The Vanin-1 (Vnn1) gene encodes a glycosylphosphati-dylinositol-linked membrane-associated pantetheinase that generates cysteamine from pantothenic acid. Studies suggest that Vanin1 may promote inflammation. Mice lacking Vnn1 showed decreased NSAID- or Schistosoma-induced intestinal inflammation, which was associated with higher glutathione levels [251]. Other evidence indicates that Vanin-1 stimulates production of inflammatory mediators by intestinal epithelial cells and thereby controls the innate immune response, possibly by antagonizing PPAR*γ* activity [252]. Epithelial Vanin-1 was also found to regulate inflammation-driven cancer development in a colitis-associated colon cancer model [253]. Evidence presented in Figure 3 demonstrates that Vnn1 likely represents a direct target gene of PPAR*α*. Expression of Vnn1 in mouse liver was markedly increased by fasting in wildtype but not PPAR*α* ^−/−^ mice (Figure 3(a)). Negligible Vnn1 expression was detected in PPAR*α* ^−/−^ mouse liver. Moreover, hepatic Vnn1 expression was significantly induced by 6 h treatment with dietary fatty acids and by the synthetic PPAR*α* agonists Wy14643 and fenofibrate (Figure 3(b)). Additional data lend strong support to the importance of PPAR*α* in Vnn1 gene regulation in small and large intestine (Figures 3(c) and 3(d)), although the results are not quite as striking as in liver. Finally, it was shown that two adjacent and partially overlapping PPREs located around 4 kb downstream of the transcription start site of the mouse Vnn1 gene were functional in a luciferase reporter assay in HepG2 cells (Figure 3(e)). PPAR*α* transfection and Wy14643 markedly increased luciferase activity, although for reasons that remain unclear, no synergism between the two treatments was observed. Overall, these data suggest that Vnn1 represents a direct PPAR*α* target gene.

The ability of PPAR*α* to stimulate fatty acid oxidation and suppress hepatic inflammation has led to the exploration of PPAR*α* agonists as a therapeutic option for nonalcohol fatty liver disease and specifically nonalcoholic steatohepatitis (NASH). Several studies in mice have shown that PPAR*α* activation can reduce or even reverse the progression of steatohepatitis [193, 254–259]. The inhibitory effect of PPAR*α* on progression of steatosis to steatohepatitis may be mediated in part by COX2 (Ptgs2), a candidate gene involved in steatohepatitis development that is suppressed by PPAR*α* [260]. In the absence of PPAR*α*, liver steatosis and inflammation are enhanced in mice chronically fed a HFD [165]. Whether the effects of PPAR*α* on NASH are primarily related to changes in hepatic TG content or occur via direct suppression of inflammatory genes and markers remains unclear. 

## 11. PPAR*α* and Biotransformation

The detoxification of endogenous and exogenous molecules is generally divided into three distinct biotransformation phases. The phase I reaction involves the introduction of a polar group into the xenobiotic molecule and is catalyzed by members of the cytochrome P450 (CYP) superfamily [179, 180, 261]. Phase II enzymes are responsible for covalent linkage of the absorbed chemicals or products of the phase I reactions with compounds such as glutathione, glucuronic acid, or amino acids and are carried out by sulfotransferases, UDP-glucuronosyltransferases (UGTs), glutathione- S-transferases (GSTs), and N-acetyltransferases [261]. The third phase corresponds to elimination of the conjugated molecule from cells and their excretion into bile or urine via specific transporters, mainly members of the superfamily ATP-binding cassette transporter proteins [262, 263]. Studies have shown that peroxisome proliferators modulate exclusively the Cyp4a class of monooxygenases (involved in the metabolism of biologically important compounds such as fatty acids, see Section 5.3) in mouse while regulating various other Cyp genes in human hepatocytes, including members of the Cyp1a, Cyp2a, Cyp2c, and Cyp2e subfamilies [24]. Our recent microarray data confirmed the human specific regulation of Cyp genes belonging to classes 1-3 by PPAR*α* in primary human hepatocytes. Interestingly, we also observed a significant induction of another subfamily member of Cyp4 enzymes, Cyp4x1, by PPAR*α* in human primary hepatocytes which was not conserved in mouse [24]. Cyp4x1 has been shown to be involved in oxidation of anandamide, which represents one of the endocannabinoids. Besides upregulation of gene expression, a number of genes involved in phase I biotransformation are downregulated by PPAR*α* in mice, including Cyp2a5, Cyp2c11, Cup2c12, and Cyp2c29 [110, 155]. 

With respect to phase II biotransformation, PPAR*α* has been shown to downregulate Glutathione-S-transferase A [GSTA], possibly leading to decreased biliary excretion of glutathione conjugates [157, 264, 265]. In contrast, expression of UDP-glucuronosyltransferase 1A (Ugt1a9), which participates with other UGT enzymes in glucuronidation of bilirubin, arachidonic, and linoleic acid metabolites, is under direct stimulatory control of PPAR*α* [158]. Overall, it is evident that PPAR*α* is a major regulator of biotransformation enzymes and governs the expression of numerous cytochrome P-450 and conjugating enzymes. However, only a small portion of the regulation seems to be conserved between rodents and humans.

## 12. Conclusion

In 2010, we are celebrating the 20th anniversary of the discovery of PPAR*α* by Isseman and Green. PPAR*α* was initially isolated as a novel nuclear hormone receptor that serves as molecular target of a diverse class of rodent hepatocarcinogens. Since then it has become clear that PPAR*α* can be activated by a large variety of endogenous and synthetic agonists including fibrate drugs. In fact, PPAR*α* is nowadays considered as a crucial fatty acids sensor that mediates the effects of numerous fatty acids and fatty acid derivatives on gene expression. Furthermore, over the years PPAR*α* has emerged as a crucial transcriptional regulator of numerous metabolic and inflammatory processes. Although PPAR*α* has mostly been connected with stimulation of fatty acid oxidation, it is now evident that the effects of PPAR*α* are much more widespread and cover numerous aspects of nutrient metabolism and energy homeostasis, including metabolism of lipoproteins, glucose/glycerol, cholesterol and bile acids, xenobiotics, and amino acids. Certainly, PPAR*α* merits the classification as a master regulator of hepatic intermediary metabolism. Until recently, much confusion surrounded the effects of PPAR*α* activation in human liver. Recent studies indicate that at least in terms of lipid metabolism, the function and specific target genes of PPAR*α* are generally well conserved between mouse and human. One of the major challenges lying ahead is to gain better understanding of the molecular mechanism underlying downregulation of gene expression by PPAR*α*, to improve insight into the specific mechanisms and pathways of endogenous PPAR*α* activation, and to better link the functional consequences of PPAR*α* activation to induction of specific PPAR*α* target genes.

## Figures and Tables

**Table 1:** *List of* *P* *P* *A* *R* *α* *target genes in different biological processes in liver.* Genes regulated by *P* *P* *A* *R* *α* in mouse are shown in lower case. Genes regulated in human and mouse are shown in ** CAPITAL BOLD**. Genes regulated only in human are shown in CAPITAL, and genes with detected functional PPRE are shown in italic font.

Lipid metabolism	Lipid/hormone transport	Adipor2 [24, 62], Cd36 [24, 62–64], **LEPR** [24, 62], *Slc27a1 *[62, 64–67], **SLC27A2** [24, 62, 63, 68], **SLC27A4** [24, 62]
	
	Acyl-CoA formation/hydrolysis/ binding	Acot1 [24, 62, 69], Acot7 [62], **ACOT12** [24, 62], ***ACSL1*** [24, 62, 70–72], **ACSL3** [24, 62], Acsl4 [24, 62, 63, 73], **ACSL5** [24, 62, 63], **ACSM3** [24, 62], Acss2 [62], ***FABP1*** [24, 62, 74, 75], Fabp2 [24, 62, 63], **FABP3** [24, 62], Fabp4 [62, 68], Fabp5 [62]
	
	Mitochondrial *β*-oxidation/oxidative phosphorylation	**ACAA2** [24, 62, 68], Acadl [24, 62, 70, 74, 76], ***ACADM*** [24, 55, 62, 70], **ACADS** [24, 54, 62, 70], **ACADVL** [24, 62, 63, 70], Acad8 [62], Acad9[62], Acad10 [62], Acot2 [24, 62, 69], Acot9 [62], Acot10 [62], ***CPT1A*** [24, 54, 55, 62, 77–79], Cpt1b [24, 62], ***CPT2*** [24, 62, 70, 80], Crat [24, 62, 74], Dci [24, 62, 65, 68, 76], Decr1 [24, 62, 76, 81], ETFA [24], Etfb [24, 62], **ETFDH** [24, 62], **HADHA** [24, 62, 63, 68, 82], **HADHB** [24, 62, 63, 68], *Hadh *[24, 62, 68, 74, 76, 83], Hadh2 [62], Hibch [24, 62], ***SLC25A20*** [24, 62, 84], **SLC22A5** [24, 62, 85, 86], **TXNIP** [24, 62], Ucp2 [24, 62, 87–89], Ucp3 [62]
	
	Ketogenesis/ketolysis	Acat1[24, 62, 68], Bdh [62], ***FGF21*** [24, 62, 90–92], Hmgcl [62], ***HMGCS2***[24, 62, 93–95]
	
	Peroxisomal *β*-oxidation	**ABCD2** [24, 62, 96], **ABCD3** [24, 62, 96], **ACAA1A** [24, 62, 68, 76], Acaa1b [24, 62], Acot3 [24, 62], Acot4 [24, 62], Acot5 [24, 62], Acot8 [62, 97], ***ACOX1*** [24, 55, 62, 68, 70, 98, 99], Crot [24, 62, 74], Decr2 [24, 62, 68, 100] **ECH1** [24, 62, 63, 68], *Ehhadh *[24, 62, 101, 102], HACL1 [24], **HSD17B4** [24, 62, 103, 104], Peci [24, 62, 65, 100], Pex11a [24]
	
	Microsomal (*ω*-hydroxylation)	ALDH3A1[24], *Aldh3a2 *[24, 62, 105], **ALDH9A1** [24], *Cyp4a1 *[74, 106–109], Cyp4a3 [55, 74, 108], ***Cyp4a10*** [24, 62, 68, 84, 106, 110], Cyp4a12a [24, 62, 65], Cyp4a14 [24, 62, 68, 110, 111], Cyp4f15 [24], Cyp4x1 [24]
	
	Lipogenesis	Acaca [62], **ACACB** [62], **AGPAT2** [62], Agpat3 [24, 62], Agpat5 [62], Agpat6 [62], Dgat1 [62, 112], ELOVL5 [62, 113, 114], **ELOVL6** [24, 62, 113, 114], Elovl7 [62], **FADS1** [24, 62, 113], *Fads2 *[62, 113, 115], Fasn [62, 112], **GPAM** [24, 62, 65], *Hsd17b12 *[62], *Lpin2* [24, 56, 62], **MLYCD** [24, 62], Mogat1 [24, 62], ***MOD1*** [24, 62, 70, 116], *Scd1 *[24, 62, 117, 118], Scd2 [62, 68], Slc25a10 [62, 100], Srebf1 [24, 62, 119]
	
	Lipases/lipid droplet proteins	***ADFP***[24, 62, 120], Ces1 [62], Ces3 [24, 62], Cidea [24, 62, 121], **CIDEC** [24, 62, 121], *Gos2 *[24, 62, 122, 123], Lipa [24, 62], Lipe [24, 62, 68], Mgll [24, 62, 63, 68], Oxpat/Lsdp5 [24, 62, 124, 125], Plin1 [24], **PNPLA2** [24, 62], **S3-12** [24, 62]
	
	Lipoprotein uptake and metabolism	***ANGPTL4***[24, 62, 126, 127], **APOA1** [128–133], *APOA2 *[24, 134], *APOA5 *[24, 135, 136], **APOCIII** [137–139], **LIPC** [24, 62], Lipg [62], *Lpl *[62, 64, 65, 140], Lrp4 [24, 62], **PCTP** [24, 62], *Pltp *[62, 65, 141, 142], Mttp [24, 62, 143], **VLDLR** [24, 62]
	
	Cholesterol/Bile transport and metabolism	**ABCA1** [24, 62, 144], **ABCB4** [24, 62, 94, 144], Abcb11 [62], Abcg5 [62, 144], Abcg8 [62, 144], Cav1 [24], ***CYP7A1*** [24, 62, 145–147], *Cyp8b1 *[62, 148], Cyp27a1 [145], FXR [62], *LXR *[144, 149], Npc1 [62], Rab9 [24, 62], Scarb2 [62], Slc10a1 [94], *Slc10a2 *[62, 150]

Other pathways	Glucose/Glycerol transport and metabolism	**AQP3** [24, 62, 151], Aqp7 [62], Aqp9 [62, 151], Fbp2 [24, 62], **G6PC** [24], *Gpd1 *[24, 62, 151], Gpd2 [62, 151], **GYK** [24, 62, 151], Gys-2 [152], Ldha [62], Pcx [62], PCK1 [24], Pdk1 [24], **PDK4** [24, 62, 153, 154]
	
	Biotransformation	**AKR1B10** [24], AKR1C3 [24], CYP1A2 [24], Cyp2a5 [110], CYP2B6 [24], CYP2C8 [24], CYP2C9 [24], Cyp2c11 [155], Cyp2c12 [155], Cyp2c29 [110], **CYP2J2** [24], CYP3A5 [24], **CYP3A7** [24], **CYP3A11** [24, 110], Cyp3a43 [24], **EPHX2** [24, 156], Gsta3 [157], MGST3 [24], ***UGT1A9*** [158]
	
	Amino Acid metabolism	**ABAT** [24, 159], Acmsd [159], **AGXT2** [24, 160], Arg1 [160], **ASL** [24, 160], Ass1 [160], **CBS** [24, 159], **CPS-1** [24, 160], Cth [159], Got1 [160], Got2 [62, 64, 160, 161], Gls [160], **GLS2** [159], **GPT** [24, 159], Hal [159], Hpd [159], **OAT** [24, 159], **ODC1** [24, 62, 159], **OTC** [24, 62, 160], **PAH** [24, 159], **PSAT1** [24, 62, 159], Tat [159, 162]
	
	Inflammation	Apcs [163], Birc3 [163], Cebpb [164], **Cd68** [24, 165], Crp [24, 163], Cxcl10/IP10 [165], **FGB** [24, 155, 166, 167], Emr1 (F4/80) [156], Icam-1 [24, 165], Ifi47 [24, 163], Igtp [163], Nfkbia [168–170], Il-1*β* [165], Il-1r1 [111], Il1rn [163], Il1rap [163], Il-6 [111, 171], Il-6ra [111, 163], Il18 [163], Lcn2 [163, 165], Lifr [163], Ccl2 [165], Ccl3 [165], Mt1 [24, 163], Mt2 [163, 165], Orm2 [163], Orm3 [24, 163], Nfkb1 [24, 164], Pla1a [24, 163], Saa2 [163], Saa4 [24, 163], Stat1 [165], Stat2 [163], Stat3 [163], Steap4 [24, 163], Stress induced protein [163], Tnf*α* [165], Traf2 [163], Vcam-1 [24, 165]
